# Supplementary material for: Experimental heatwaves compromise sperm function and cause transgenerational damage in a model insect
Source: Nat Commun. 2018 Nov 13;9:4771. doi: 10.1038/s41467-018-07273-z (PMC6233181; doi:10.1038/s41467-018-07273-z)
Supplement: Supplementary file 3 — Supplementary Information [file 41467_2018_7273_MOESM3_ESM.pdf]

SUPPLEMENTARY INFORMATION - NCOMMS-18-24197-T

Experimental heatwaves compromise sperm function and cause transgenerational damage in a model insect

Sales *et al.*

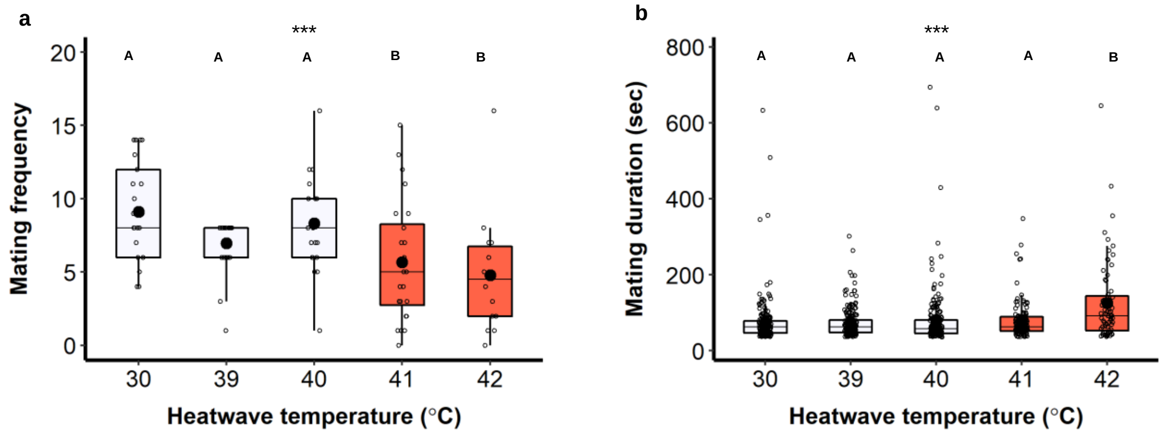

Supplementary Figure 1| Mating behaviour of males following exposure to increasing 5-day heatwave temperatures. Orange boxes indicate heatwave treatment conditions for the species. Successful matings defined as >35s of unbroken copulation. **a,** Mating frequencies across 60-minute mating trials. Sample sizes from left to right: n_{males} = 25, 24, 21, 24, 14. **b,** Mating durations. Sample sizes from left to right: $n_{matings}$ = 178, 183, 174, 119, 67 where each data point is a mating event. Boxplots display a mean dot, median line, interquartile range (IQR) boxes, 1.5*IQR whiskers and data points. Significance thresholds: ***, P<0.001 letters denote differences to the 30 °C treatment. Raw data are available in the associated Source Data file.

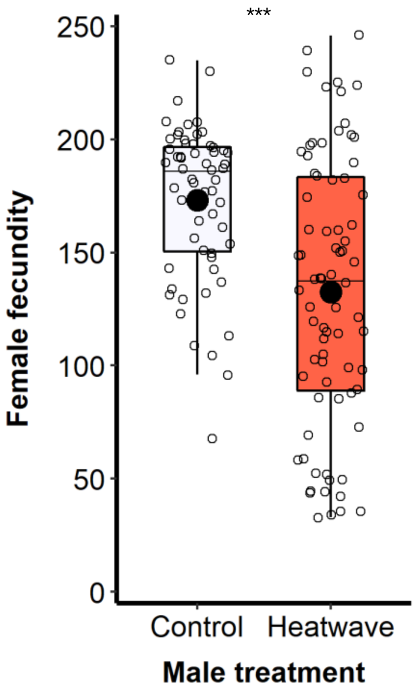

Supplementary Figure 2| Effect of male heatwaves on female post-mating fecundity. Number of eggs laid by females over 10 days after mating to control (white, *n*=59) or heatwave-treated (orange, *n*=76) males. Boxplots display a mean dot, median line, interquartile range (IQR) boxes, 1.5*IQR whiskers and data points. Significance thresholds: ***, $P<0.001$. Raw data are available in the associated Source Data file.

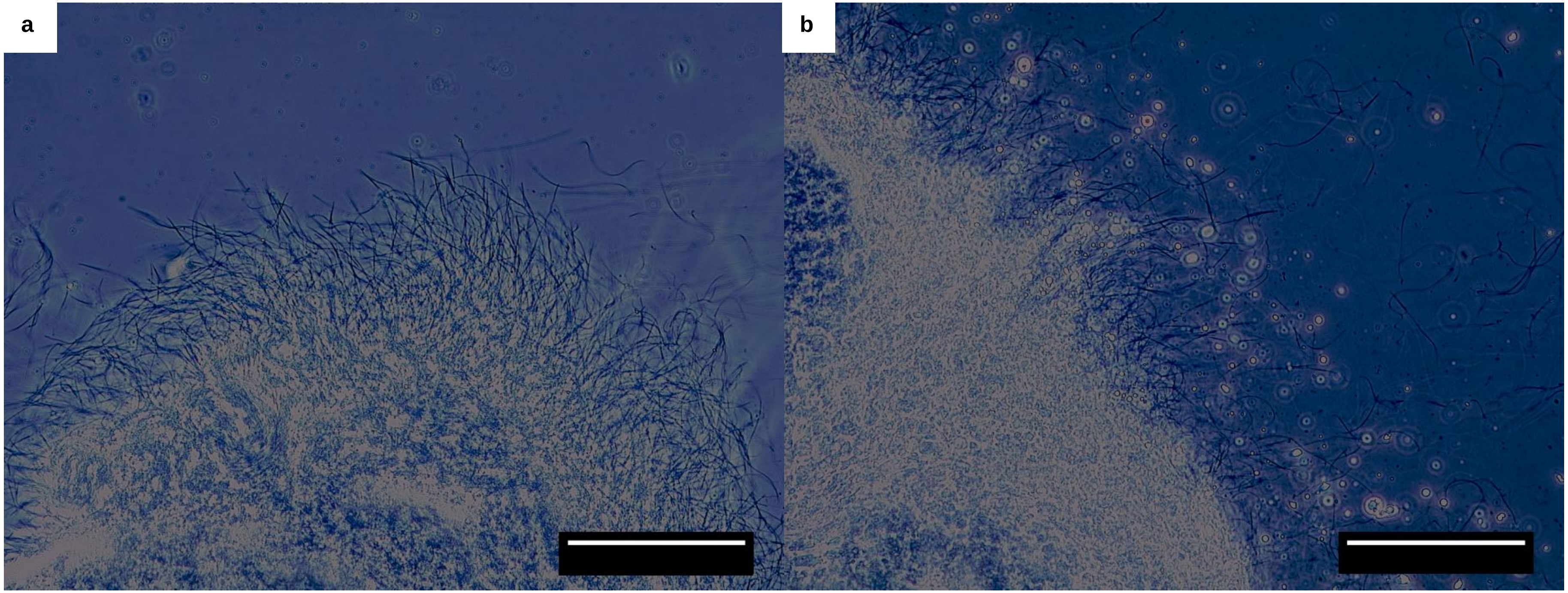

Supplementary Figure 3| Effect of heatwaves on spermatophore ejaculate integrity. Phase contrast images of sp erm masses released from spermatophores inseminated into females by males after exposure to 5-day (a) 30ºC control or (b) 42ºC heatwave conditions. Scale bars are 100 microns long.

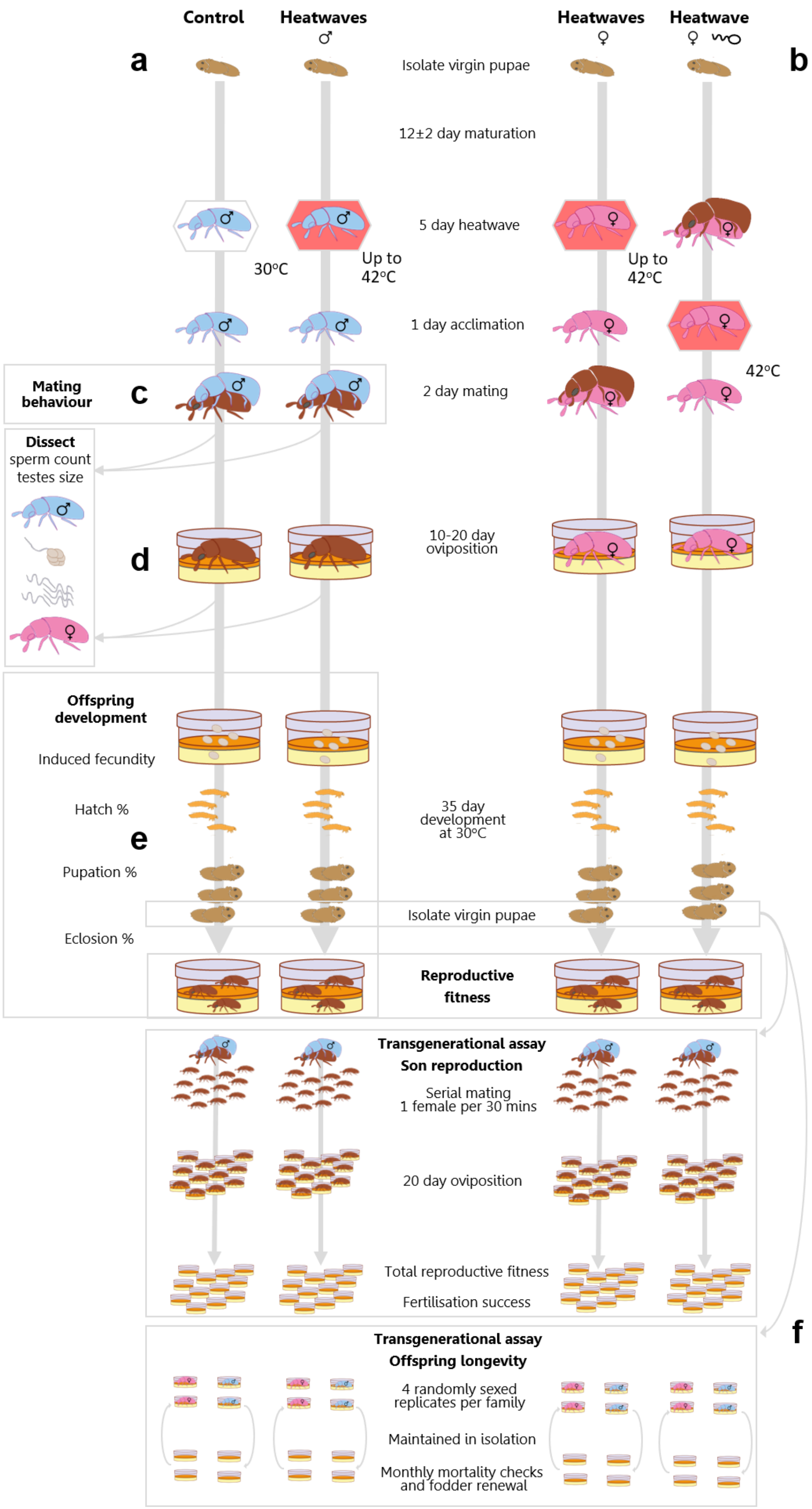
Control
Heatwaves ♂
Heatwaves ♀
Heatwave ♀ ♂
a
b
Isolate virgin pupae
12±2 day maturation
5 day heatwave
30°C
Up to 42°C
Up to 42°C
1 day acclimation
42°C
Mating behaviour
c
2 day mating
Dissect
sperm count
testes size
d
10-20 day oviposition
Offspring development
Induced fecundity
Hatch %
e
Pupation %
Eclosion %
35 day development at 30°C
Isolate virgin pupae
Reproductive fitness
Transgenerational assay
Son reproduction
Serial mating
1 female per 30 mins
20 day oviposition
Total reproductive fitness
Fertilisation success
f
Transgenerational assay
Offspring longevity
4 randomly sexed replicates per family
Maintained in isolation
Monthly mortality checks and fodder renewal

Supplementary Figure 4 | Summarised protocols from several heatwave assays. a. Virgin male and female adults, **b.** mated females. All treated individuals were paired to control mates. **c.** Mating behaviour. **d.** Following mating, males and females were dissected for ejaculate count and survival assays, with the majority of females isolated in fodder to oviposit. **e.** In the male treatments, the offspring development through different life stages was recorded by periodic resampling. In all treatments, reproductive success was assessed by counting adult offspring production. **f.** During pupation a subset of offspring were taken to assess transgenerational effects. One son per parental replicate from each treatment was tested following mating opportunities with a series of 13 unmated females (30 minutes each) to compare male reproductive fitness. In the paternal heatwave treatments, the longevity of four, randomly sexed sibling offspring per replicate cross was measured.

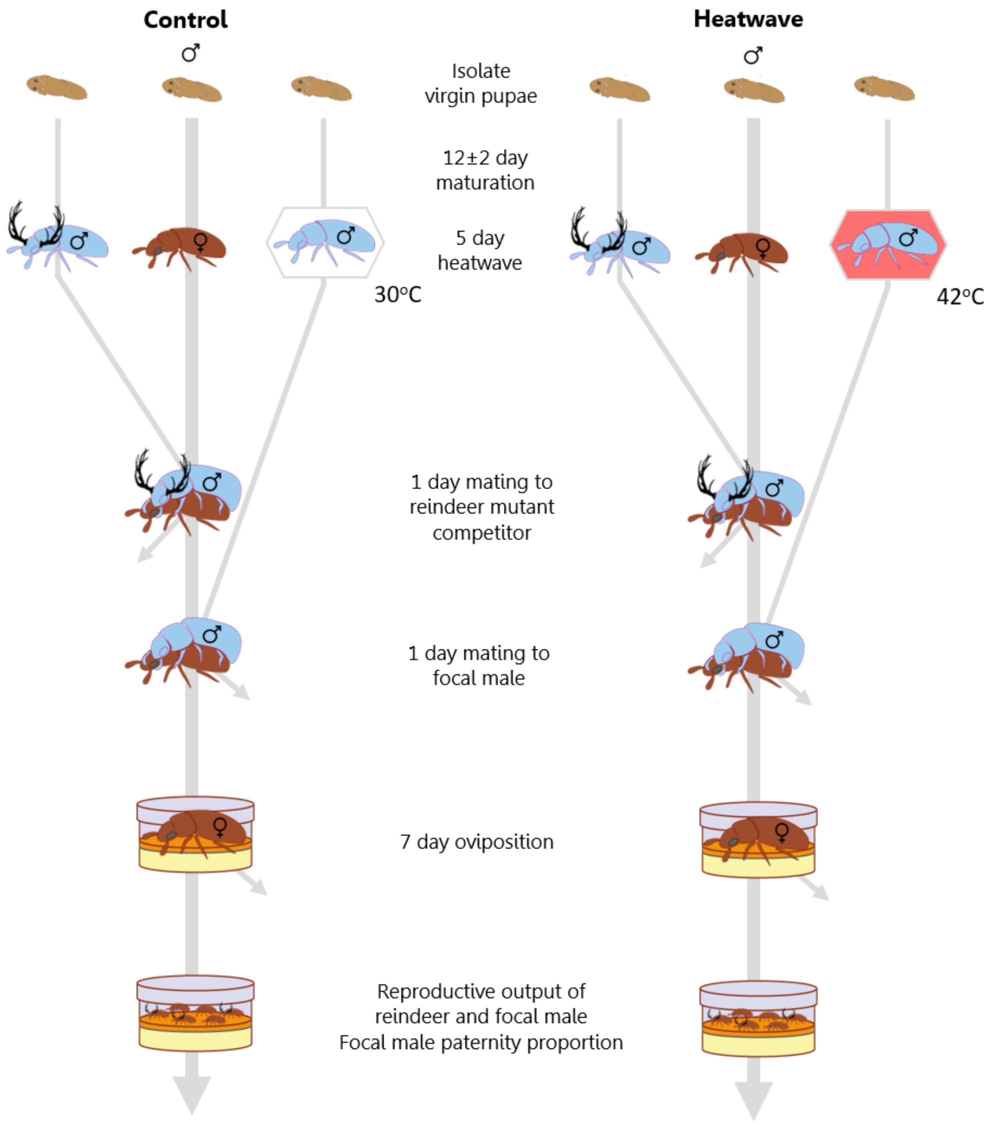

Supplementary Figure 5 | Protocol to assess sperm competitiveness following heatwaves. Females were mated to competitor males carrying a homozygous dominant 'Reindeer' (*Rd*) mutation with a clubbed antennae phenotype. Females were then paired with either a control or heatwave treated wild type phenotype male. Reproductive success of each male was measured by scoring number of offspring with either clubbed (*Rd*) or wild type antennae following 7 days of female oviposition.

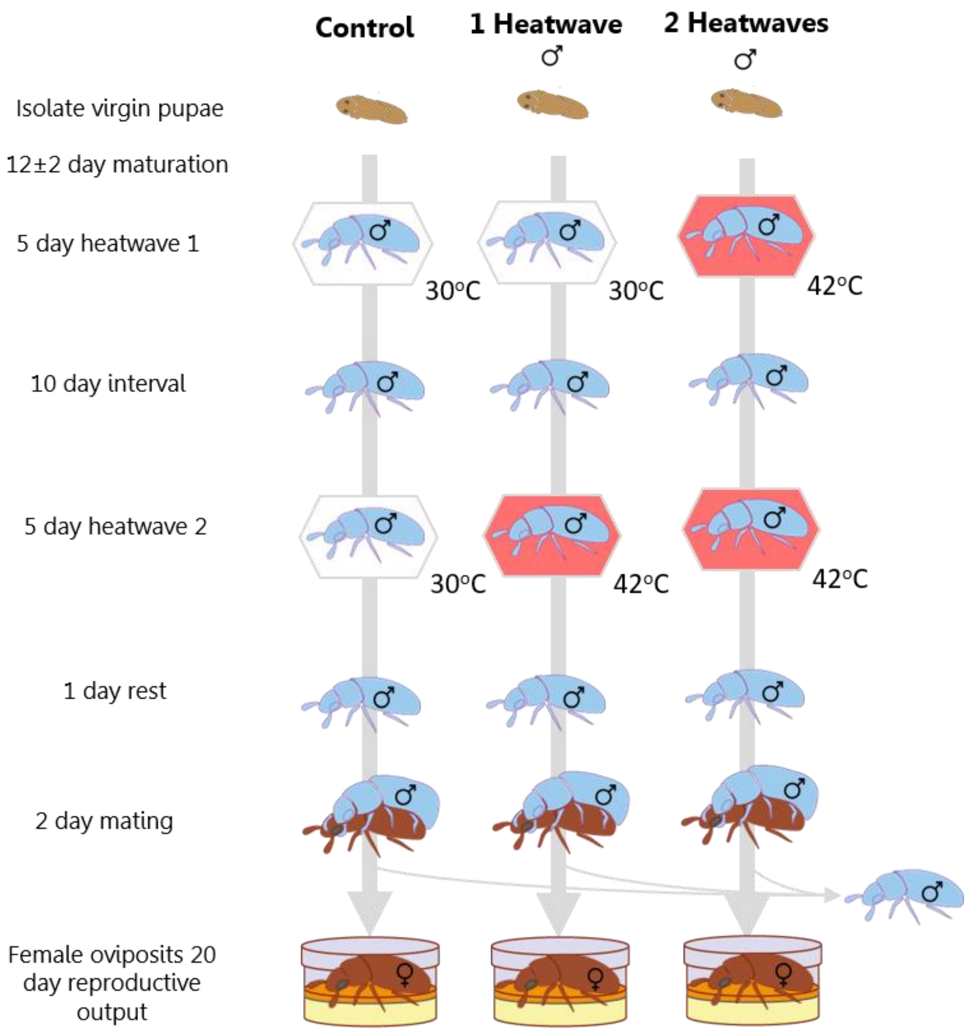

Supplementary Figure 6 | Protocol for the response of males to single versus double heatwaves. Males were exposed to either control, single heatwaves or double heatwave treatments, and placed with a control female a two-day mating period. Males were then removed and reproductive output measured as offspring production across 20 days of female oviposition.

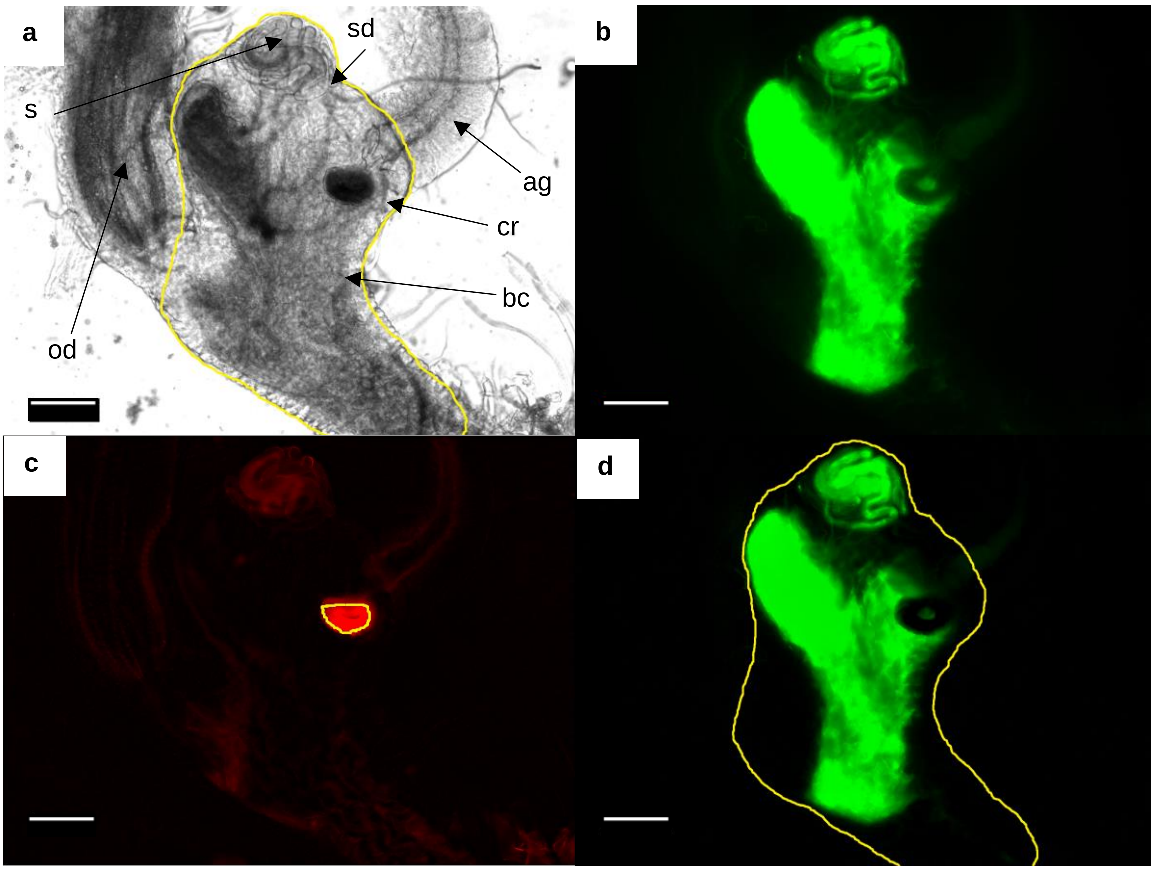

Supplementary Figure 7| Image processing and analysis of sperm distribution in the female reproductive tract. a. Brightfield image of female reproductive tract with region of interest (ROI) outlined in yellow drawn around the perimeter walls of the tract. Identified structures: (ag) accessory gland, (bc) bursa copulatrix, (cr) chitin ring, (od) oviduct, (s) spermatheca, (sd) spermathecal duct. **b.** Fluorescence captured in the green channel, including contributions from green fluorescence protein (GFP) tagged sperm heads and general auto-fluorescence (AF). **c.** Structural (non-sperm) AF captured in the red channel. An additional ROI is drawn around areas displaying clear AF; the chitin ring at the base of the spermathecal duct typically exhibits high autofluorescence. The mean fluorescence intensity in this ROI was measured in both **b** (Int_{GFP}) and **c** (Int_{AF}). **d.** The image in **c** was multiplied by the correction factor, $Int_{GFP} \div Int_{AF}$, and the resulting image subtracted from **b** to leave only GFP-sperm-derived fluorescence as evident in **d.** Scale bars are 100 microns long.

Experiment	Figure	Factor	Analysis of deviance (with/without factor) χ^2 / F statistic	Degrees of freedom (numerator, denominator)	P value from drop1{stats} (Refs 66,82,83)	Structure, error family (link)	R^2 for GLMs pseudo-R^2 (Ref 66) for GLMMs r.squaredGLMM{MuMIn} (Ref 95)	Factor levels (treatments)	Sample size	Mean ± SE	Model betas	Model t / z value	Model P-values summary (model) (Refs 84,85)
Male and female reproductive fitness	1	Heatwave temperature x Sex	χ = 40.2	5, 537	<0.001	quasi poisson (log)	21	Female 30°C	75	210.9 ± 8.9	5.35		
		Heatwave temperature	χ = 71.7	5, 537	<0.001			Male 30°C	79	191.8 ± 6.3	0.19	2.7	0.008
		Sex	χ = 51.2	1, 541	<0.001			Female 35°C	34	254.9 ± 8.9	0.21	3.2	0.002
								Male 35°C	33	258.3 ± 9.8	0.08	1.1	0.292
								Female 38°C	43	259.7 ± 11.2	0.1	1.4	0.149
								Male 38°C	48	187.1 ± 9.7	0	-0.1	0.959
								Female 39°C	35	227.8 ± 15	-0.09	-1.6	0.118
								Male 39°C	43	193.8 ± 10.9	0.1	1.1	0.287
								Female 40°C	35	234.2 ± 14.3	-0.23	-2.4	0.017
								Male 40°C	48	150.9 ± 10.4	-0.07	-0.6	0.517
								Female 42°C	28	210 ± 14.2	-0.34	-3.3	0.001
								Male 42°C	42	106.8 ± 14.4	-0.58	-4.8	<0.001
Stored sperm reproductive fitness	2a	Female stored sperm heatwave treatment	χ = 14.1	1, 115	<0.001	negative binomial (log)	10	Female 42°C sperm 30°C	55	163 ± 6.3	5.09		
								Female 42°C sperm 42°C	62	108.8 ± 6.1	-0.41	-3.9	<0.001
Paternity proportion in competition	2b	Male heatwave treatment	χ = 97.3	1, 114	<0.001	quasi binomial (logit)	44	Male 30°C	65	0.77 ± 0.02	1.21		
								Male 42°C	51	0.33 ± 0.04	-1.99	-9.3	<0.001
Impacts of a second heatwave on male reproductive fitness	2c	Male heatwave treatment	χ = 25.2	2, 82	<0.001	negative binomial (log)	21	Male 30°C	20	227.4 ± 26.8	5.42		
								Male 42°C	35	125.9 ± 20.1	-0.59	-1.2	0.24
								Male 2*42°C	29	14.3 ± 5.2	-2.76	-5.3	<0.001
Sperm count	3a	Male heatwave treatment	χ = 7.2	1, 93	0.007	negative binomial (log)	6	Male 30°C	38	6.0 ± 1.0 $*10^4$	11		
								Male 42°C	56	1.6 ± 0.4 $*10^4$	-1.33	-2.7	0.008
Sperm distribution in female storage	3b	Male heatwave treatment	F = 19.3	1, 45	<0.001	gaussian (log)	31	Male 30°C	22	2703.1 ± 377	7.9		
								Male 41°C	24	905.6 ± 184.2	-1.1	-3.3	0.002
Sperm viability	3c	Male heatwave treatment	χ = 10.1	1, 25	<0.001	GLMM(count effect) binomial (logit)	marginal 27 / conditional 46	Male 30°C	10	0.82 ± 0.04	1.87		
								Male 42°C	16	0.31 ± 0.06	-4.33	-3.5	<0.001
Egg hatch	3h	Male heatwave treatment	χ = 17.9	1, 69	<0.001	quasi binomial (logit)	20	Male 30°C	39	0.85 ± 0.03	1.86		
								Male 42°C	32	0.41 ± 0.08	-1.54	-4.1	<0.001
Larval development	3h	Male heatwave treatment	χ = 3.1	1, 54	0.074	quasi binomial (logit)	8	Male 30°C	38	0.99 ± 0	4.39		
								Male 42°C	18	0.96 ± 0.02	-0.79	-1.8	0.072
Pupal eclosion	3h	Male heatwave treatment	χ = 0.9	1, 54	0.334	binomial (logit)	3	Male 30°C	38	1 ± 0	7.33		
								Male 42°C	18	1 ± 0	-0.76	-0.1	0.32
Egg to adult success	3h	Male heatwave treatment	χ = 19.3	1, 69	<0.001	quasi binomial (logit)	21	Male 30°C	39	0.84 ± 0.03	1.77		
								Male 42°C	32	0.40 ± 0.07	-1.51	-4.3	<0.001

Supplementary Table 1| Extended model summaries for all statistical analyses for Figures 1, 2 and 3. Includes overall significance of factors of interest, model structure, treatment group means and standard error, model betas and associated significance values.

Experiment	Figure	Factor	Analysis of deviance (with/without factor) χ^2 / F statistic	Degrees of freedom (numerator, denominator)	P value from drop1{stats} (Refs 66,82,83)	Structure, error family (link)	R^2 for GLMs pseudo-R^2 (Ref 66) for GLMMs r.squaredGLMM{MuMIn} (Ref 95)	Factor levels (treatments)	Sample size	Mean ± SE	Model betas	Model t / z value	Model P-values summary (model) (Refs 84,85)
Offspring longevity (weeks)	4a, b	Paternal heatwave treatment	χ = 4.7	1,55	0.03	Accelerated failure time survival, gaussian	NA	Father 30°C	28	66.1 ± 2.5	2.67		
								Father 40°C	29	57.8 ± 2.9	-8.29	-2.2	0.027
Reproductive fitness of sons	4c	Paternal heatwave treatment	χ = 9.7	2, 88	<0.001	GLMM(block effect) gaussian (identity)	marginal 10 / conditional 13	Father 30°C	48	1254.8 ± 62.7	1244		
								Father 42°C	42	960.4 ± 68.7	-302.18	-3.3	0.002
Mating success by sons	4d	Paternal heatwave treatment	χ = 13.2	2, 88	<0.001	GLMM(block effect) poisson (log)	marginal 14 / conditional 14	Father 30°C	48	6.7 ± 0.3	1.91		
								Father 42°C	42	4.9 ± 0.3	-0.32	-3.6	<0.001
Reproductive fitness of sons	4e	Female stored sperm heatwave treatment	χ = 19.8	2, 101	<0.001	quasipoisson (log)	14	Mother 30°C	27	1113.4 ± 88.4	7.02		
								Mother 42°C sperm 30°C	42	877 ± 51.6	-0.24	-2.1	0.035
								Mother 42°C sperm 42°C	34	632.9 ± 77.3	-0.56	-4.4	<0.001
Mating success by sons	4f	Female stored sperm heatwave treatment	χ = 14.4	2, 101	<0.001	poisson (log)	10	Mother 30°C	27	6 ± 0.5	1.79		
								Mother 42°C sperm 30°C	42	5.7 ± 0.3	-0.05	-0.5	0.631
								Mother 42°C sperm 42°C	34	4.1 ± 0.4	-0.39	-3.4	<0.001
Mating frequency	S1a	Male heatwave temperature	χ = 36.9	4, 103	<0.001	poisson (log)	16	Male 30°C	25	9.1 ± 0.7	2.21		
								Male 39°C	24	7.0 ± 0.4	-0.27	-2.7	0.008
								Male 40°C	21	8.3 ± 0.7	-0.09	-0.9	0.369
								Male 41°C	24	5.7 ± 0.8	-0.48	-4.4	<0.001
								Male 42°C	14	4.8 ± 1.1	-0.64	-4.6	<0.001
Mating duration	S1b	Male heatwave temperature	F = 14.3	4, 717	<0.001	gamma (identity)	7	Male 30°C	178	75.8 ± 5.0	75.8		
								Male 39°C	183	72.7 ± 2.9	-3.1	-0.5	0.623
								Male 40°C	174	80.6 ± 6.2	4.8	0.7	0.477
								Male 41°C	119	78.9 ± 4.5	3.2	0.4	0.671
								Male42°C	67	125.7 ± 13.1	49.9	3.8	<0.001
Female fecundity	S2	Male heatwave temperature	χ = 15.2	1, 133	<0.001	negative binomial (log)	9	Male 30°C	59	173.1 ± 4.5	5.15		
								Male 42°C	76	132.6 ± 6.8	-0.27	-3.9	<0.001

Supplementary Table 1 (cont'd)| Extended model summaries for all statistical analyses for Figure 4, and Supplementary Figures 1 and 2. Includes overall significance of factors of interest, model structure, treatment group means and standard error, model betas and associated significance values.
